# Bacterial cell biology outside the streetlight

**DOI:** 10.1111/1462-2920.13406

**Published:** 2016-07-15

**Authors:** Silvia Bulgheresi

**Affiliations:** ^1^ Department of Ecogenetics & Systems BiologyUniversity of ViennaAlthanstrasse 14Vienna1090Austria

## Abstract

As much as vertical transmission of microbial symbionts requires their deep integration into the host reproductive and developmental biology, symbiotic lifestyle might profoundly affect bacterial growth and proliferation. This review describes the reproductive oddities displayed by bacteria associated – more or less intimately – with multicellular eukaryotes.

“We become what we behold. We shapeour tools and then our tools shape us”

Culkin, J.M. (1967). A schoolman's guide to Marshall McLuhan. Saturday Review, pp. 51–53, 71–72.

## Introduction

According to rough estimates, less than 0.1% of the up to 10^9^ bacterial species thriving on planet Earth may be cultivated. Yet the reproduction of only a handful of lab‐reared bacteria is being studied (Goley, 2013). The current quest to unravel the mechanisms underlying bacterial reproduction is therefore comparable to a drunkard's search. Its inherent observational bias – the so‐called *streetlight effect* – is treacherous as it may easily generate wrong assumptions or even beliefs. Here, I wish to review cell biological studies on bacterial symbionts, some of which already confuted well‐rooted tenets of bacterial cell growth and division (Table 1). I will use the term symbiont *sensu lato*, that is to say every organism living together with a differently named organism (de Bary, 1879). Traditionally, symbionts are classified in ecto‐ and endosymbionts depending on whether they are outside or inside the host body respectively. In this review, although formally situated inside the host, I will regard the gut epithelium as superficial and consider its inhabitants – the gut microbiota – together with other ectosymbionts. Owing to space limitations, I will not review systems in which the molecular mechanisms underlying anomalous symbiont growth have not been investigated yet. These are the vast majority and include – for example – the ectosymbionts of unicellular (Desai *et al*., 2010; Strassert *et al*., 2010; Brune, 2014; Brune and Dietrich, 2015) and colonial eukaryotes (Bright *et al*., 2014), the endosymbionts of unicellular eukaryotes (Schulz and Horn, 2015) and of abyssal bathymodiolin mussels *Candidatus* Endonucleobacter bathymodioli (Zielinski *et al*., 2009). Finally, also due to space limitations, I will be able neither to review our knowledge about mitochondrial and plastid reproduction and its control by the eukaryotic cell, nor to consider bacteria whose cell biology and physiology is dramatically affected by developmental programs [e.g., elementary body–reticulate body transition in *Protchlamidiae* associated to free‐living amoebae (Horn, 2008; Schrallhammer and Schweikert, 2009)]. And now, before I embark on describing abnormal reproductive modes, I must briefly summarize how conventional bacteria grow and divide.

**Table 1:** Examples of bacterial symbionts displaying non‐canonical division modes

Host	Symbiont	Environment surrounding the symbiont	Reproductive anomaly	Presence of FtsZ	Host factor(s) affecting symbiont division
***Extracellular symbionts***					
*Laxus oneistus* nematode	*Candidatus* Thiosymbion oneisti (*Gammaproteobacteria*)	Marine sediment	Widening and symmetric longitudinal fission	YES	NN
*Eubostrichus fertilis* nematode	*Eubostrichus fertilis* nematode ectosymbiont (*Gammaproteobacteria*)	Marine sediment	Cell elongation (up to 45 µm) and symmetric transverse fission	YES	NN
*Eubostrichus dianeae* nematode	*Eubostrichus dianeae* nematode ectosymbiont (*Gammaproteobacteria*)	Marine sediment	Cell elongation (up to 120 µm) and symmetric transverse fission	YES	NN
*Epulopiscium* spp.	Surgeonfish (Firmicutes)	Gastrointestinal tract	Multiple intracellular offspring		
*Metabacterium polyspora*	Guinea pig (Firmicutes)	Gastrointestinal tract	Multiple endospore formation or binary fission	YES	NN
*Mus musculus*	Segmented Filamentous Bacterium (SFB; Firmicutes)	Gastrointestinal tract	Cell elongation, segmentation and intracellular offspring or sporulation	YES	NN
***Intracellular symbionts***					
Legume	Rhizobia (*Alphaproteobacteria*)	Modified plant cell (nodule) in nodule	Cell elongation (block of cytokinesis)	YES	Nodule‐specific cysteine‐rich peptides (NCRs)
Weevil	*Sodalis pierantonius*	Modified insect cell (bacteriocyte) in bacteriome	Cell elongation (block of cytokinesis)	YES	Coleoptericin A (ColA)
Aphid	*Buchnera aphidicola* *(Gammaproteobacteria)*	Modified insect cell (bacteriocyte) in bacteriome	Gigantism (block of cytokinesis)	YES	Secreted cysteine‐rich proteins?
	*Sulcia muelleri*	Modified insect cell (bacteriocyte) in bacteriome	Gigantism (block of cytokinesis)	YES	NN

## Bacterial growth and division for dummies

**Figure 1:**
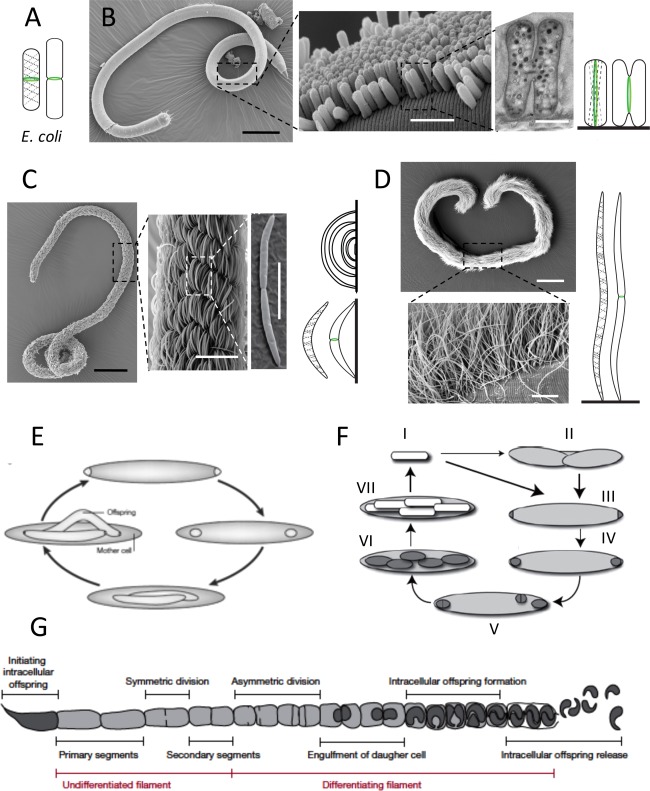
(A) Drawing of a pre‐divisional (left) and a dividing (right) *Escherichia coli* cell displaying non‐constricted and constricted FtsZ rings (green), respectively, and dashed lines indicating localization of the cell wall synthesis coordinator MreB. (B–D) Spatial arrangement and division modes of three stilbonematid nematode symbionts; (B) Scanning electron micrographs of *Laxus oneistus* (juvenile stage, leftmost panel), its bacterial coat (middle panel), and one dividing ectosymbiont (rightmost panel), and drawings of a non‐dividing and of a dividing ectosymbiont. Scale bars are 100 µm, 3 µm, and 500 nm, from left to right. (C) Scanning electron micrographs of *Eubostrichus fertilis* (leftmost panel), its bacterial coat (middle panel), and one dividing ectosymbiont (rightmost panel), and drawings displaying how long crescent‐shaped symbiont cells overlie shorter ones (top), and a non‐dividing and a dividing ectosymbiont. Scale bars are 100, 20, and 5 µm, from left to right. (D) Scanning electron micrographs of *E. dianeae* (top) and of its bacterial coat (bottom), and drawing of a non‐dividing and a dividing ectosymbiont. Scale bars are 200 µm (top) and 10 µm (bottom). In all nematode ectosymbiont drawings FtsZ rings are displayed in green, dashed lines indicate hypothetical localization of the MreB protein and black lines the host cuticle; symbionts are not drawn to scale. Scanning electron micrographs by Nikolaus Leisch and drawings by Nika Pende. (E) The life cycle of *Epulopiscium* spp. Polar, FtsZ‐based division produces two daughter cells that are engulfed by the mother cell. Eventually, growth of the offspring overtakes that of the mother cell until the latter deteriorates and the offspring emerge from the weakened mother cell envelope (slightly modified from Angert (2005)). (F) The life cycle of *M. polyspora* divided into seven stages (I‐VII). Mature endospores of *M. polyspora* (I) are ingested by a guinea pig. Once these have reached the small intestine, they germinate. Whereas a minority undergoes binary fission (II), the majority undergoes bipolar division (III). As *M. polyspora* proceeds into the caecum, the polar forespores are engulfed (IV). Fully engulfed, early forespores may still undergo binary fission (V) and elongate (VI). As the forespores develop into mature spores (VII), the mother cell lyses and the cycle begins anew (I) (from Ward and Angert 2008). (G) Schematic representation of an SFB filament highlighting stages of its growth and differentiation from Schnupf *et al*. (2015).

A wealth of exhaustive and well‐written reviews is available on the fundamental process of bacterial reproduction [among the most recent ones: Bramkamp and Van Baarle (2009), Young (2010); den Blaauwen (2013); Chang and Huang (2014); Rowlett and Margolin (2015)]. The peptidoglycan (PG or murein) wall or sacculus confers physical strength and defines the shape of most bacteria (Egan and Vollmer, 2013; de Pedro and Cava, 2015; Randich and Brun, 2015). It overlies their cell membrane and consists of a mesh‐like macromolecule of glycan chains that are cross‐linked together via peptide chains. In model rods, two protein assemblies (the elongasome and the divisome) direct the modification and synthesis of the cell wall: during growth, the elongasome inserts freshly synthetized PG along the length of the rod, and during septation a cytokinesis complex called the divisome (Nanninga, 1991) completes the steps of constriction and new PG synthesis at midcell (Typas *et al*., 2012). Both divisome and elongasome consist of scaffolding cytoskeletal‐like proteins (cytoplasmic), inner membrane spanning proteins and a profusion of periplasmic enzymes including PG synthases and hydrolases. In nearly all bacteria, the tubulin‐like GTPase FtsZ regulates the localization and activity of divisome components. In canonical rod‐shaped bacteria, which divide by transverse binary fission, spatiotemporal regulation of the divisome is fairly understood. During division, the GTP‐dependent polymerization of FtsZ creates a ring‐shaped structure called the Z‐ring at the centre of the cell (Fig. 1A) and perpendicular to the axis of chromosome segregation (Bi and Lutkenhaus, 1991; Löwe and Amos, 1998; Mukherjee and Lutkenhaus, 1998; Erickson *et al*., 2010). The Z‐ring is attached to the cytoplasmic face of the membrane via membrane‐associated proteins and recruits ten essential proteins important for septum assembly and Z‐ring constriction (Hale *et al*., 1997; Pichoff and Lutkenhaus, 2005). In *Escherichia coli*, the mechanism for targeting the Z‐ring to midcell involves the Min system, a machinery that inhibits the formation of the contractile ring at the cell poles (Raskin and de Boer, 1999; Hale *et al*., 2001; Monahan *et al*., 2014), and nucleoid occlusion (NO), which prevents nucleoid guillotining by the Z‐ring (Woldringh *et al*., 1991, 1990; Bernhardt and de Boer, 2005). Much less is known about the spatiotemporal regulation of the elongasome, for which the actin‐like protein MreB appears to be the major scaffold for coordinating PG precursor synthesis and polymerization (Fig. 1; Esue *et al*., 2005, 2005; Salje *et al*., 2011; Ozyamak *et al*., 2013).

Although the bacteria treated in the following sections are coccoid, rod‐shaped or filamentous and therefore, morphologically, are not significantly different from the conventional ones, they display at least one of the following peculiarities: (i) polymorphism, often within the same population, (ii) reproduction modes other than transverse binary fission and/or (iii) alternation between two different reproduction modes.

## Dressed to cooperate: growth anomalies of ectosymbionts

Non‐conventional reproductive modes seem to be the specialty of the house in the *Stilbonematinae*, a small family of mesopsammic (interstitial) roundworms: *Laxus oneistus* nematodes are covered by a single layer of rod‐shaped bacteria tightly packed with one another, and standing perpendicularly to the worm's surface as to form a columnar epithelium (Fig. 1B); the filamentous ectosymbionts of two other stilbonematid nematodes, *Eubostrichus fertilis* and *Eubostrichus dianeae*, are attached to the worm cuticle with two or one pole(s) respectively (Fig. 1C and D; Pende *et al*., 2014). The first one forms a bacterial coat resembling a braided rope, the second one resembling a fur. All the stilbonematid symbionts molecularly characterized fall in the Marine Oligochaete and Nematode Thiotrophic Symbionts (MONTS) cluster (Polz *et al*., 1994; Bayer *et al*., 2009; Heindl *et al*., 2011; Pende *et al*., 2014), more recently referred to as *Candidatus* Thiosymbion (Zimmermann *et al*., 2016). This is a basal group of *Gammaproteobacteria* related to free‐living sulfur‐purple bacteria of the *Chromatiaceae*. Candidatus Thiosymbion spp. may synthesize organic carbon compounds by exploiting the energy released by the oxidation of reduced sulfur (Polz *et al*., 1994; Hentschel *et al*., 1999; Musat *et al*., 2007; Bayer *et al*., 2009). We proved that the *L. oneistus*‐associated rods grow in width, set constricting Z‐rings parallel to their long axes and divide longitudinally by default (Fig. 1B; Leisch *et al*., 2012). Remarkably, the newly described Z‐ring appears not only 90° shifted with respect to model rods, but also elliptic and highly discontinuous. As for the symbionts of *E. fertilis* and *E. dianeae*, they reproduce by setting and constricting a single Z‐ring transversally, at midcell (Fig. 1C and D, respectively; Pende *et al*., 2014). Strikingly, though, in the former symmetric FtsZ‐based fission occurs in crescents with lengths from 4 to 45 µm leading to an unprecedented cell length variation of one order of magnitude within the same population (Pende *et al*., 2014). This suggests that cell size may not be the primary trigger of division in these bacteria. As for the *E. dianeae* symbiont, despite lengthening up to 120 µm, it forms and constricts a single FtsZ ring at midcell, which makes it the longest unicellular organism known to divide by symmetric transverse division (Pende *et al*., 2014). We identified the *min* operon in *Laxus* and *Eubostrichus* symbionts, but its role in septum positioning is still unknown. Moreover, although we identified the *mre* operon in all the aforementioned stilbonematid symbionts we do not know how MreB coordinates cell wall growth in the nematode ectosymbionts. Finally, we must determine the exact number of genomes that get symmetrically localized and their orientation and segregation mechanisms. Do the different symbiont spatial dispositions, i.e., the different symbiont reproductive strategies, represent adaptations to different nematode hosts or to different nutritional regimes (Vadia and Levin, 2015)? Did *L. oneistus* symbiont longitudinal fission or did *E. fertilis* symbiont bipolar attachment evolve to favour symbiont vertical transmission? Are these two symbionts metabolically more dependent on their host than the *E. dianeae* symbiont, which can afford to let one daughter cell detach from the host surface?

We hope that omics‐based comparisons among stilbonematids occupying different habitats or carrying different types of bacterial coats will clarify whether the latter serve specific, host‐symbiont metabolic networks or physiological interdependencies, that – in turn – evolved as adaptations to specific habitats.

## Outside but inside: fish gut residents

Although extraordinarily long, the *E. dianeae* symbiont is not the longest known. The size record holders are indeed the surgeonfish intestinal symbionts *Epulopiscium* spp. Their heterotrophic cigar‐shaped cells are up to 600 × 80 µm and reproduce by forming at least two intracellular daughter cells (Fig. 1E; Angert *et al*., 1993). Cells colonizing the gut of a given host fish synchronize internal offspring initiation and development so that offspring always grows during the day, concomitantly with host feeding. Reproduction begins as two Z‐rings form at the mother cell's poles. As the rings fully constrict, two polar cells form, are engulfed by the mother cell and grow in a membrane‐bound cytoplasmic compartment until they completely fill the mother cell. Finally, the mother cell undergoes a form of programmed cell death that likely conserves the biochemical resources accumulated during growth (Ward *et al*., 2009) and offspring emerge through a split in the mother cell envelope. Notably, despite developmental synchrony, inhabitants of a single population vary up to five times in volume (Mendell *et al*., 2008). How can *Epulopiscium* spp. accommodate growth of internal offspring? Although their genome size is unexceptional (ca. 4 Mb), each cell contains up to 10^5^ copies (Mendell *et al*., 2008). However, in contrast to most bacteria, in which chromosomes are distributed throughout the cytoplasm, *Epulopiscium* nucleoids are located at the cell periphery, which may also allow *Epulopiscium* to respond promptly to environmental stimuli.

## Outside but inside: mammalian gut residents

Another curious gastrointestinal dweller is *Metabacterium polyspora*. Unlike most endospore formers, this guinea pig gut resident produces up to nine endospores per mother cell (Chatton and Pérard, 1913; Robinow, 1957). Although no *Metabacterium*‐like symbiont has been maintained in culture, morphologically similar symbionts have been found in various rodent species (Kunstyr *et al*., 1988). The natural life cycle of *M. polyspora* (Fig. 1F) requires the bacterium to cycle through the gastrointestinal tract and therefore relies on the coprophagous nature of the guinea pig for survival (Angert and Losick, 1998). Only mature endospores survive passage through the mouth and stomach of the host, and may germinate in the small intestine. Here, some cells undergo binary fission, but most cells begin to sporulate (Angert and Losick, 1998). From the small intestine, *M. polyspora* cells are deposited in the guinea pig caecum where they complete sporulation. Cells with engulfed forespores or mature endospores do not seem to undergo binary fission and, after traversing the lower intestine, they are finally eliminated from the host with its feces. If a guinea pig ingests the defecated spores, the life cycle starts again. The process of endospore formation in *M. polyspora* differs from that of prototypical endospore‐forming bacteria such as *Bacillus subtilis* (Angert and Losick, 1998). The asymmetric cell division of *M. polyspora* normally takes place at both cell poles (rather than at one only) and DNA is partitioned into both polar compartments and it is also retained in the mother cell. Therefore, unlike sporulating *B. subtilis*, which is diploid, *M. polyspora* must contain three or more genomes, which makes coordinating DNA replication and segregation with offspring formation even more challenging (Angert and Losick, 1998). After engulfment, the forespores can undergo division to produce multiple forespores that grow and mature into endospores. Most endospore‐forming Firmicutes produce a single, dormant, invulnerable spore only to survive adverse environmental conditions or to increase their dispersal. It is therefore stunning that the reiteration of such a developmental program is part of the normal *M. polyspora* life cycle. The coordination of multiple endospore formation with transit through the gut, combined with a coprophagous natural host, might favour this reproduction mode over binary fission. Additionally, the occasional experience of harsh conditions outside the host would still trigger *M. polyspora* to initiate sporulation for survival and dispersal.

There is another group of low‐GC Gram‐positive bacteria that forms endospores for reproduction and dispersal in a very unorthodox way: the segmented filamentous bacterium (SFB) or *Candidatus* Arthromitus (Fig. 1G). SFB reside in the intestinal tracts of many vertebrate species such as mice and ourselves (Klaasen *et al*., 1992; Yin *et al*., 2013; Schnupf *et al*., 2015) and have received much interest because of their ability to educate the gut immune system and to induce a healthy level of physiological inflammation (Schnupf *et al*., 2013; Ivanov *et al*., 2009). Already four decades ago, ultrastructural studies of murine gut SFB supported the following life cycle: attachment to epithelial cells via the holdfast tip of the so‐called “initiating intracellular offspring” leads to SFB embedding among the microvilli followed by filamentous growth. A complex developmental program thereupon starts at the distal tip and ultimately leads to intracellular offspring formation and release (Davis and Savage, 1974; Chase and Erlandsen, 1976; Ferguson and Birch‐Andersen, 1979). According to this model, when filaments grow longer than 50 µm in length (most filaments are ≈100 μm and can be up to 1 mm long), the large primary filament segments start to undergo a symmetrical division to form smaller secondary segments. These differentiate by dividing asymmetrically to form a mother and a daughter cell. The latter becomes engulfed and subsequently divides to form two intracellular offspring within the surrounding mother cell segment. Intracellular offspring are then released from the filament by breakdown of the filament septa and reattach to the host. Remarkably, the intracellular offspring have two possible fates, either holdfast‐producing differentiation, or maturation to form a spore. In the first case, the active offspring are released into the lumen of the intestine and they attach to the intestinal epithelium to establish new filaments within the host. In the second case, maturation results in two intracellular offspring cells that are encased in a common spore coat, which forms an endospore. As the cells of the intestinal epithelium are constantly shed and renewed, they are not a stable substrate. Therefore, the production of intracellular offspring released from the dying parental filament probably evolved to allow SFB to reposition itself inside the same host. Additionally, the endospore provides an effective alternative for the SFB to disperse. These alternative forms of offspring (either active or dormant) allow the SFB to maintain stable populations within a given host and to colonize new hosts after surviving harsh environments such as aerobic ones or the highly acidic upper gut. The proposed SFB life cycle was recently and completely recapitulated *in vitro* (Schnupf *et al*., 2015). This is exciting, as it will permit the investigation of the complex developmental stages of SFB and the detailed dissection of the unique SFB–host interaction at the cellular and molecular levels.

### What controls the growth of gut residents?

The host‐secreted molecules that determine SFB cell fate, as well as those putatively controlling that of other gut residents are not known. Where should we look for those? It is a challenge for the gut to stably host 100 trillion bacteria belonging to more than 100 different species without this inducing inflammation or without the host falling victim of pathogenic infections (estimates by Qin *et al*., 2010). Perturbations in intestinal homeostasis are the basis of various diseases such as obesity, diabetes and inflammatory bowel disease. Given that immune homeostasis relies on several, partly overlapping immunological mechanisms we still have not fully grasped how it is achieved (Hooper and Macpherson, 2010). However, several effectors of the innate immune system controlling the number of specific gut residents have already been identified and these are antimicrobial peptides (AMPs). This should not come as a surprise as the growing consensus is that both pathogenic and mutualistic bacteria share similar microbe associated molecular patterns (MAMPs) and trigger an immune response. Nevertheless, its outcome differs between pathogenic and mutualistic associations: in the former, the immune response triggers the elimination of deleterious microbes, in the latter a molecular dialogue is initiated which results in homeostasis and immunotolerance (Nussbaum and Locksley, 2012). In a mutualistic context, antimicrobial peptides produced by the epithelia contribute to maintaining the population structure of the microbiota and prevent these from penetrating the underlying tissues. The epithelia are covered with a thin mucus layer that is kept nearly sterile, while microbes abound in the above‐lying lumen (Hooper and Macpherson, 2010). In the small intestine, this is accomplished by the Paneth cells which secrete into the gut AMPs and other antimicrobial proteins thereby limiting contact between the microbiota and epithelial tissues (Kobayashi *et al*., 2005; Vaishnava *et al*., 2008). In addition, AMPs such as α‐defensins also regulate the composition of the microbiota in the lumen (Salzman *et al*., 2003, 2010; Wilson *et al*., 1999). In particular, bacteria known as *Firmicutes* decreased with higher defensin production by Paneth cells. Similarly, Vaishnava *et al*. (2011) showed that the antimicrobial lectin RegIII‐γ prevents any contact between the mouse commensal microbiota and the epithelial surface of the small intestine. Disruption of this physical separation, by knocking out the RegIII‐γ gene, resulted in bacterial proliferation at the intestinal epithelial surface, which subsequently triggered an adaptive immune response against the microbiota. Secretion of the RegIII‐γ protein was shown to inhibit bacterial growth within a band extending 50 µm from the epithelium, creating a “no‐microbe's‐land” where no commensal can grow or stimulate the host immune system. Given that RegIII‐γ is not expressed in the large intestine, this is one of the first cases describing the involvement of the innate immune system in the regulation of mutualism via a local immune response. Also in the fruit flies gut a well‐adjusted level of AMP expression under healthy, as well as pathogenic conditions is essential for maintaining homeostasis (Ryu *et al*., 2008).

In conclusion, a respectable body of data indicates that vertebrate‐secreted AMPs target and regulate the number of gut micro‐residents. Although their capacity to affect the morphology or reproduction mode of commensals has not been shown yet, this is likely, as research reviewed in the following section indicates.

## “Staying put”: reproductive anomalies of endosymbionts with non‐reduced genomes

**Figure 2:**
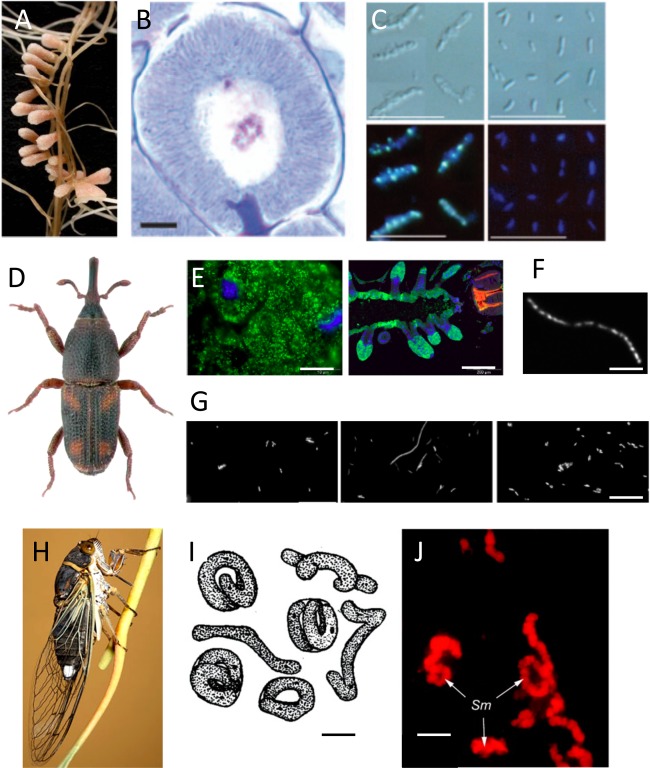
(A) *Medicago truncatula* nodules (http://www.isv.cnrs-gif.fr). (B) A *M. truncatula* symbiotic cell entirely filled with terminally differentiated, strongly elongated bacteroids; scale bar is 10 mm; from Kereszt *et al*. (2011). (C) Size, shape, and DNA content of *S. meliloti* bacteroids isolated from nitrogen‐fixing *M. truncatula* nodules (left) and free‐living, cultured *Synorhizobium meliloti* bacteria (right). Nomarski (top) and fluorescence (bottom) microscopy of DAPI stained bacteria and bacteroids. Scale bars are 10 µm; from Mergaert *et al*. (2006). (D) A *Sitophilus* weevil; http://www.pestid.msu.edu/insects-and-arthropods/grain-weevils/. (E) *Sodalis pierantonius* endosymbionts within the weevil bacteriocytes (left) and the bacteriome of adult mesenteric caeca (right); scale bars are 10 µm (left) and 200 µm (right); from Login and Heddi (2013). (F) Stained chromosomes of a *S. pierantonius* cell; scale bar is 20 µm; from Login *et al*. (2011). (G) Effect of low concentrations of ColA and ColB on *E. coli* morphology. Bacteria were incubated in LB broth (left, control), in LB with 8 mM ColA (middle, cell gigantism), or in LB with 8 mM ColB (right, no cell gigantism observed); scale bar is 20 µm; from Login *et al*. (2011). (H) The cicada *Diceroprocta semicincta* feeding on plant xylem sap; credit: Adam Fleishman. (I) Reproduction of drawing of symbionts dissected from the bacteriome of the cicada *Philaenus spumarius*; scale bar is 10 µm; from Moran *et al*. (2005). (J) Fluorescence in situ hybridization targeting the *Bacteroidetes* symbiont *Candidatus* Sulcia muelleri (Sm) dissected from the bacteriomes of the cicada *Clastoptera arizonana*; scale bar is 10 µm; from Moran *et al*. (2005).

If cell gigantism and associated polyploidy have been observed in numerous microbial symbionts, only in those of legumes and weevils the molecular triggers, i.e., the host‐secreted molecules that block bacterial cytokinesis have been nailed (Van der Velde *et al*., 2010; Login *et al*., 2011). In most cases, the benefit of plant and insect symbionts is privileged acquisition of nutrients and a growth niche. Whether the symbionts reside extracellularly, in luminal spaces between cells and tissues, or live an intracellular existence they are closely associated with the host cells. When legumes interact with nitrogen‐fixing rhizobia, the symbiosis leads to formation of new organs, the root nodules (Fig. 2A). These organs house millions of endosymbiotic rhizobia. Within symbiotic nodule cells, these *Alphaproteobacteria* become capable of reducing atmospheric nitrogen to ammonium, which is transferred to the plant and used for its growth. These irreversibly differentiated rhizobia (also referred to as bacteroids; Fig. 2B) have altered physiology and metabolism. In some legumes, as in the model plant *Medicago truncatula*, bacteroids have increased membrane permeability, highly amplified genome content and – whether elongated or branched – are much larger than soil‐dwelling rhizobia. These bacteroids are incapable of cell division and reproduction (Mergaert *et al*., 2006; Maroti *et al*., 2011). Although this terminal differentiation of bacteroids is not observed in all legumes and is therefore not essential *per se* for symbiotic nitrogen fixation, it improves the symbiotic efficiency of the bacteroids (Oono and Denison, 2010). In *M. truncatula*, symbiotic nodule cells produce nodule‐specific AMPs of a particular family called NCR for nodule‐specific cysteine‐rich peptides (NCRs; Mergaert *et al*., 2003; Alunni *et al*., 2007). Remarkably, the *M. truncatula* NCR gene family consists of several hundred genes, which are all specifically expressed in *Rhizobium*‐infected nodule cells. The NCRs are responsible for the terminal differentiated state of the endosymbiont *Sinorhizobium meliloti* in *M. truncatula* nodules (Fig. 2C; Van der Velde *et al*., 2010). Similarly to other plant and animal AMPs, they effectively kill both Gram‐positive and Gram‐negative bacteria *in vitro* at prototypical concentrations. NCRs are transported via exocytosis to the bacteroids and some of the NCRs enter the bacterial cytosol and likely have intracellular bacterial targets. The *in vivo* and *in vitro* effects of NCRs on *S. meliloti* are dramatically different. The peptides quickly kill the rhizobia *in vitro*, whereas bacteroids do not grow but maintain active metabolism. The difference between the *in vivo* and *in vitro* effect of NCRs could be explained if we assume that, *in vivo*, several tens or hundreds of native peptides, each likely present at very low concentrations, have a different effect that a single, highly concentrated peptide applied *in vitro*. Moreover, particular conditions prevalent in nodules, such as the low free oxygen concentration, needed for activity of the oxygen‐sensitive nitrogenase, could modulate the bacterial responses to the NCRs in such a way that the bacteroids survive without growing. Some NCRs inhibit bacterial division *in vivo* and *in vitro*, leading to cell elongation. Such NCRs were localized at the division site of *S. meliloti* cells, and may therefore interfere with the bacterial cytokinetic machinery. However, the high sequence variability of NCRs suggests diversity in their functions, mode of actions and targets involved in different aspects of bacteroid metabolism, but could also be an adaptation to the high diversity of soil rhizobia. Analysis of the sequence of NCRs showed that they are subject to diversifying evolution, which is compatible with such a hypothesis (Alunni *et al*., 2007). Moreover, it is remarkable and yet unexplainable that most plants contain hundreds of genes encoding for cysteine‐rich proteins similar to innate immunity‐dedicated AMP genes (Silverstein *et al*., 2007), even if they do not employ them to form symbioses or to defend themselves. The NCRs are indeed expressed only in nodules and are not induced during treatment of *M. truncatula* with pathogens.

In conclusion, legumes may adopt effectors of the innate immune system to dominate their endosymbionts in order to maximize their own profits. In a striking case of convergent evolution, this is also the case for *Sitophilus* weevils (Fig. 2D–G; Login *et al*., 2011, Login and Heddi, 2013). Cereal weevils house *Sodalis pierantonius* (Fig. 2F; Oakeson *et al*., 2014; formerly known as *Sitophilus* Primary Endosymbiont, or SPE) permanently, in the female germ cells from which they are transmitted to the progeny. Early during embryogenesis, *S. pierantonius* induces the differentiation of bacteriocyte cells that form the bacteriome (Heddi *et al*., 1999), a specific organ that secludes endosymbionts (Fig. 2E; Anselme *et al*., 2008). These host cells, in response, express an adapted response in order to maintain the symbionts within the bacteriome (Anselme *et al*., 2008; Login *et al*., 2011). Sequestering symbiotic bacteria in the bacteriome – where only a few immune effectors are expressed – protects them from exposure to‐ and elimination by a standard, non‐tailored systemic immune response. This humoral response does indeed attack *S. pierantonius*, when this bacterium is injected in the insect hemolymph (Nakabachi *et al*., 2005; Ratzka *et al*., 2011). In the weevil bacteriome, instead, Coleoptericin A (ColA) is the only antimicrobial expressed constitutively and colA transcript levels correlate with *S. pierantonius* density (Login and Heddi, 2012; Anselme *et al*., 2008; Login *et al*., 2011; Masson *et al*., 2015). While ColA exhibits bactericide activity against Gram‐negative bacteria and kills them at high concentrations, it exhibits a bacteriostatic activity against *S. pierantonius* at the low concentrations likely existing in the bacteriocytes. Microscopic observations showed that inhibition of cytokinesis by ColA led to filamentation, namely to up to 50 µm‐long *E. coli* cells (Fig. 2G) and up to 200 µm‐long *Nardonella* cells, the primary endosymbiont of the weevil *Rhynchophorus ferrugineus*. Additionally, it was also showed that *S. pierantonius* reduced significantly in size *in vivo* following functional knockout of the *col*A gene (Login *et al*., 2011). However, whether this bacterial size reduction is due to a resumption of bacterial cytokinesis or a multiplication of small bacteria remains unclear. *In vivo* silencing of the *col*A gene also resulted in an extensive dispersion of the symbiont outside of the bacteriome, which strongly supports the idea that, besides its morphology, ColA controls symbiotic bacteria through a specific and local inhibition of bacterial cytokinesis. How does ColA block bacterial fission? Without affecting the eukaryotic cell, ColA might interact with bacterial outer membrane protein C (OmpC) and OmpA and, in the bacterial cytoplasm, with the chaperonin GroEL. groEl gene deletion led to filamentation due to the misfolding of FtsE (Susin *et al*., 2006; Fujiwara and Taguchi, 2007). Considering that both ColA peptide activity and groEL gene deletion induce filamentation, it was proposed that ColA hampers cell division through inhibition of the GroEL chaperonin activity. The presence of accessible hydrophobic patches is the major feature guiding the interaction of GroEL with its substrate. The hydropathy plots showed that ColA N‐terminus is positively charged and it might therefore form stable complexes with GroEL. Interestingly, the interaction of ColA with GroEL is extremely specific since no interaction was detected with other eukaryotic chaperones, which may explain why ColA does not harm weevil bacteriocytes (Login *et al*., 2011).

## The reproductive minimalists: reproduction of intracellular endosymbionts with highly reduced cytokinetic and/or cytoskeletal machineries

Could AMPs‐induced cytokinesis also keep at bay symbionts with extremely reduced genomes? This question is not easy to answer given that many of these not only lack divisome genes but also a canonical PG. Like weevils, aphids carry an obligatory mutualistic endosymbiont, *Buchnera aphidicola*, which harbours from 20 to several hundreds genome copies, varying from cell to cell (Komaki and Ishikawa, 1999, 2000). Notably, the genomic copy number of this gammaproteobacterium is low in aphid embryos, increases during postembryonic development to adulthood, and decreases during insect ageing. Moreover, aphids have two different morphs. While the aphid colony usually consists mostly of apterae, the wingless morphs, some environmental cues increase the population of alatae, the winged morphs. Alatae‐associated *Buchnera* have twice as many genomic copies per cell as apterae‐associated ones. The amplification of the genomic DNA by *Buchnera* might be a genetic counterbalance against accumulating mutations drift, which leads to long‐term massive genome reduction (Moran, 1996; Baumann *et al*., 1996; Andersson and Kurland, 1998; Fares *et al*., 2002). This low genome copy number of *Buchnera* in aphid embryos might result from the elimination of mutated copies during symbiont transmission from mother to progeny. During host postembryonic development, DNA replication not followed by cytokinesis restores the high number of genomic copies of *Buchnera* characteristic of adult host insect. Apart from the copy number, also the physical conformation of the *Buchnera* genome varies in response to the physiological state of the host insect (Komaki and Ishikawa, 2000).

We do not know the molecular mechanisms that link host physiology with the number of *Buchnera* genome copies per cell. Curiously, a novel class of genes that encodes small, secreted, often cysteine‐rich, proteins appears to be transcribed in bacteriocytes (Shigenobu and Stern, 2013). These genes are first expressed in developing aphids, exactly when the prospective bacteriocytes engulf the symbionts, and bacteriocyte‐specific expression is maintained throughout the aphid's life. The expression pattern suggests that recently evolved secretion proteins act within bacteriocytes, perhaps to mediate the symbiosis with beneficial bacterial partners, which is reminiscent of the aforementioned leguminous plant NCRs (Shigenobu and Stern, 2013).

Polyploidy has also been suspected (Wu *et al*., 2006) and subsequently proven (Woyke *et al*., 2010) for the Bacteroidetes *Sulcia muelleri*, another sap‐feeding insect endosymbiont (Fig. 2H–J). This was estimated to contain 180–880 genome copies per cell. As in the case of *Buchnera*, it is not known what controls the number of *Sulcia* genomes or cells.

How do obligate endosymbionts reproduce without a cell wall? Since the original report of Klieneberger in 1935 (Klieneberger, 1935) cell wall‐deficient bacteria (CWDB or L‐forms) have been described many times in the literature. Molecular genetic analysis of the L‐form variant of *B. subtilis* showed that conversion into a form that can replicate reasonably efficiently in the absence of a cell wall requires only two genetic changes (Leaver *et al*., 2009). Remarkably, despite the limited mutational changes required, L‐form cells completely abandon the normally essential cell division machinery used by virtually all extant bacterial cells, and proliferate instead by a mechanism of membrane tubulation or blebbing referred to as extrusion‐resolution. This process is, at least for *B. subtilis*, completely independent of the cell wall precursor synthetic pathway and the major cytoskeletal proteins, MreB and FtsZ. A recent report on *Listeria* L‐forms described vesiculation, a process involving similarly complex membrane dynamics (Dell'Era *et al*., 2009). A wide range of bacteria is thought to be able to enter the L‐form state, including both Gram‐positive and ‐negative lineages (Domingue and Woody, 1997). Modern extant cells may have retained L‐form production as a back‐up process in case of cell wall defective synthesis or damage. These eventualities are likely ancient, given the widespread production of PG active antibiotics, such as β‐lactams, glycopeptides and lipopeptides, by various primitive groups of bacteria (Goodfellow and Fiedler, 2010; Gupta, 2011). Consistently, a considerable body of experimental and clinical evidence supports the pathogenicity of CWDB (Domingue and Woody, 1997). Probably all known bacterial species can be converted to L‐forms by a variety of inducing agents. Among the best known of such agents are cell wall‐inhibiting antibiotics, high concentrations of amino acids, and peptidases and PG lytic enzymes. It is therefore not surprising that (i) obligate PG‐deficient endosymbionts can reproduce and that (ii) L‐form bacteria have also been found to form non‐pathogenic symbioses with a wide range of plants, where they confer resistance against subsequent challenge by bacterial pathogens (Paton, 1987). Examples include the systemic protection by L‐forms of the pathogen *Pseudomonas syringae* of bean plants against halo‐blight (Amijee *et al*., 1992) and the protection of cabbage against *Xanthomonas campestris* (Waterhouse *et al*., 1996). More recently, L‐forms of the endophyte *Bacillus amyloliquefaciens* have been observed in vanilla crops where they likely protect them from diseases (White *et al*., 2014).

But between bacterial symbionts bearing complete cytokinetic machinery and L‐forms other exotic cases have been reported. Chlamydiae are important pathogens and symbionts lacking the cell‐division protein FtsZ but it was recently shown that some environmental ones have cell wall sacculi, albeit consisting of a novel PG type (Pilhofer *et al*., 2014, Jacquier *et al*., 2015). The discovery of chlamydial PG challenges the current hypothesis that it is the absence of a cell wall, to make FtsZ non‐essential.

## Concluding remarks and open questions

The recent extension of cell biological studies to the microbial symbioses field already confuted a number of tenets well‐rooted in this discipline, underscoring the dangers of the so‐called *streetlight effect*. Even considering the few systems discussed in this non‐systematic – and therefore not exhaustive – review only, it is obvious that many more long‐standing cell biological dogmas will be broken. More generally, the study of cell wall growth, divisome assembly and positioning, and chromosome segregation in organisms displaying atypical growth modes will help us to elucidate what lies at the core of the bacterial cytokinetic machinery and how it evolved. This information is most precious as it can be easily exploited to design new antibiotics, thereby filling the present “discovery void” (Silver, 2011, 2014; Li and Ma, 2015). Of note, *Gammaproteobacteria* are not only the most common microorganisms associated with animals (Sachs *et al*., 2011), but they also include several common, still challenging pathogens.

Besides informing cell biology and biomedicine, by study how symbionts divide we can learn whether (and possibly how) – in evolutionary time – the associative lifestyle shaped bacterial reproduction. As symbiont morphologies and spatial dispositions defy easy explanations, the host role or that of abiotic factors in shaping them is an open question. For example, the *L. oneistus* symbiont (Leisch *et al*., 2012) and, possibly, that of *Kentrophoros* (Fenchel and Finlay, 1989) divide longitudinally allowing both daughter cells to keep contact with the host surface. Nevertheless, longitudinal division was also suggested for endosymbionts of the gutless oligochaete *Olavius* (Giere and Krieger, 2001; reviewed in Bright and Giere, 2005) and of the deep‐sea mussel *B. puteoserpentis* (Zielinski *et al*., 2009) although these, as endosymbionts, unlikely evolved this fission mode to better transmit the associative lifestyle to both their offspring.

Unfortunately, many symbionts, including the vast majority of the ones mentioned here are not cultivable yet. Moreover, we ignore how their respective free‐living counterparts, if existing, divide. It is possible that they either retained or lost their capacity to switch between canonical and non‐canonical reproduction depending on their free‐living or symbiotic condition. More efforts are necessary to cultivate these symbionts or – at least – to characterize the morphology and reproduction mode of their environmental counterparts, if existing.

Finally, not to incur into yet another observational bias by merely moving from one streetlight to the next, it is necessary to extend cell biological investigations to other environmental organisms from both the Archaea and Eukarya domains (Bernander *et al*., 2011), as well as other ecological niches such as extreme environments. Only by bringing cell biology outside the streetlight we can identify conserved mechanisms of cell growth and reproduction and find new ways to block it for biomedical purposes.
